# Long non-coding HCG18 promotes intervertebral disc degeneration by sponging miR-146a-5p and regulating TRAF6 expression

**DOI:** 10.1038/s41598-017-13364-6

**Published:** 2017-10-16

**Authors:** Yanhai Xi, Tingwang Jiang, Weiheng Wang, Jiangming Yu, Yang Wang, Xueming Wu, Yunfei He

**Affiliations:** 1 Department of Spine Surgery, Changzheng hospital, Second Military Medical University, Shanghai, 200003 China; 2 Department of Immunology and Microbiology, Institution of Laboratory Medicine of Changshu, Changshu, 215500 Jiangsu China

## Abstract

Intervertebral disc degeneration (IDD) is associated with the deterioration of nucleus pulposus (NP) cells due to hypertrophic differentiation and calcification. Emerging studies have shown that long noncoding RNAs (lncRNAs) play critical roles in the development of IDD. Using bioinformatics prediction, we hereby sought to identify the lncRNAs that regulate the expression of microRNA-146a-5p (miR-146a-5p), an IDD-related inflammatory factor. Our study demonstrated that lncRNA HCG18 acted as an endogenous sponge to down-regulate miR-146a-5p expression in the NP cells by directly binding to miR-146a-5p. In addition, HCG18 expression was up-regulated in the patients with IDD, bulging or herniated discs, and its level was positively correlated with the disc degeneration grade. *In vitro*, miR-146a-5p up-regulation HCG18 retarded the growth of NP cells by decreasing S phase of cell cycle, inducing cell apoptosis, recruitment of macrophages and hypercalcification. Conversely, down-regulation of miR-146a-5p exerted opposite effects. Furthermore, we elucidated that TRAF6, a target gene by miR-146a-5p, was modulated by HCG18 expression. Restore of TRAF6 expression by virus infection reserved the effect of HCG18 on the NP cells. Altogether, our data indicated that HCG18 suppressed the growth of NP cells and promoted the IDD development via the miR-146a-5p/TRAF6/NFκB axis.

## Introduction

Intervertebral disc degeneration (IDD), the major cause of low-back pain, affects the majority of the population, with roughly 10% turning to be chronically disabled^1,2^. The high prevalence of IDD not only impacts on the quality of life in IDD patients, but also presences a severe burden to public health^3^. Although IDD is generally regarded as a natural process of disc aging, many patients show accelerated disc degeneration due to environmental and genetic factors^4,5^. IDD is characterized by the reduced number of nucleus pulposus (NP) cells, degradation of proteoglycans, aggrecan and collagen in extracellular matrix (ECM) content, leading to disrupt the homeostasis of the NP in the disc and shift disc maintenance towards a degenerative and catabolic state^6^. Furthermore, the osteogenic differentiation of the NP cells is involved in IDD development^7^. Emerging studies have demonstrated that a series of cellular events are involved in IDD, ranging from variations in inflammatory cytokines to dysregulation of matrix synthesis^8^. However, the dysregulation of gene expression is a very complex process, and the molecular mechanisms of IDD have still not been fully elucidated.

LncRNAs, a group of regulatory RNAs longer than 200 nucleotides, are linear RNA transcipts of the mammalian genome without protein-coding function^9^. LncRNAs have unique characteristics such as high tissue specificity and low sequence conservation. Although lncRNAs don’t code for any protein, they still play important roles in physiological and pathological processes, including genome imprinting, gene expression regulation, cellular differentiation, and nuclear-cytoplasmic trafficking^10,11^. LncRNAs could serve as “molecular sink” to regulate RNA or protein by chromatin modification, pre-mRNA splicing, and mRNA degradation^12,13^. Recently, accumulating evidences have shown that aberrantly expressed lncRNAs play a role in the IDD process. Understanding of lncRNAs in IDD may contribute greatly to the understanding of the molecular mechanisms of IDD progression.

Our previous study has demonstrated that microRNA-146a-5p (miR-146a-5p) inhibits macrophages recruitment and protects the NP cells from TNF-α-induced apoptosis by targeting TRAF6. We hereby aimed to explore the potential lncRNA that sponging miR-146a-5p in IDD. Firstly, bioinformatics prediction was utilized to identify the lncRNA. The results showed that HCG18 modulated the miR-146a-5p expression in the NP cells, and the elevated HCG18 was found in patients with IDD or herniated disc. Next, we assessed the role of HCG18 on the proliferation and apoptosis of the NP cells, osteogenic differentiation, and macrophages recruitment. Our findings suggested that HCG18 serves as a stimulus in the development of IDD by targeting miR-146a-5p.

## Results

### HCG18 functions as miR-146a-5p sponge and is up-regulated in IDD patients

**Figure 1:**
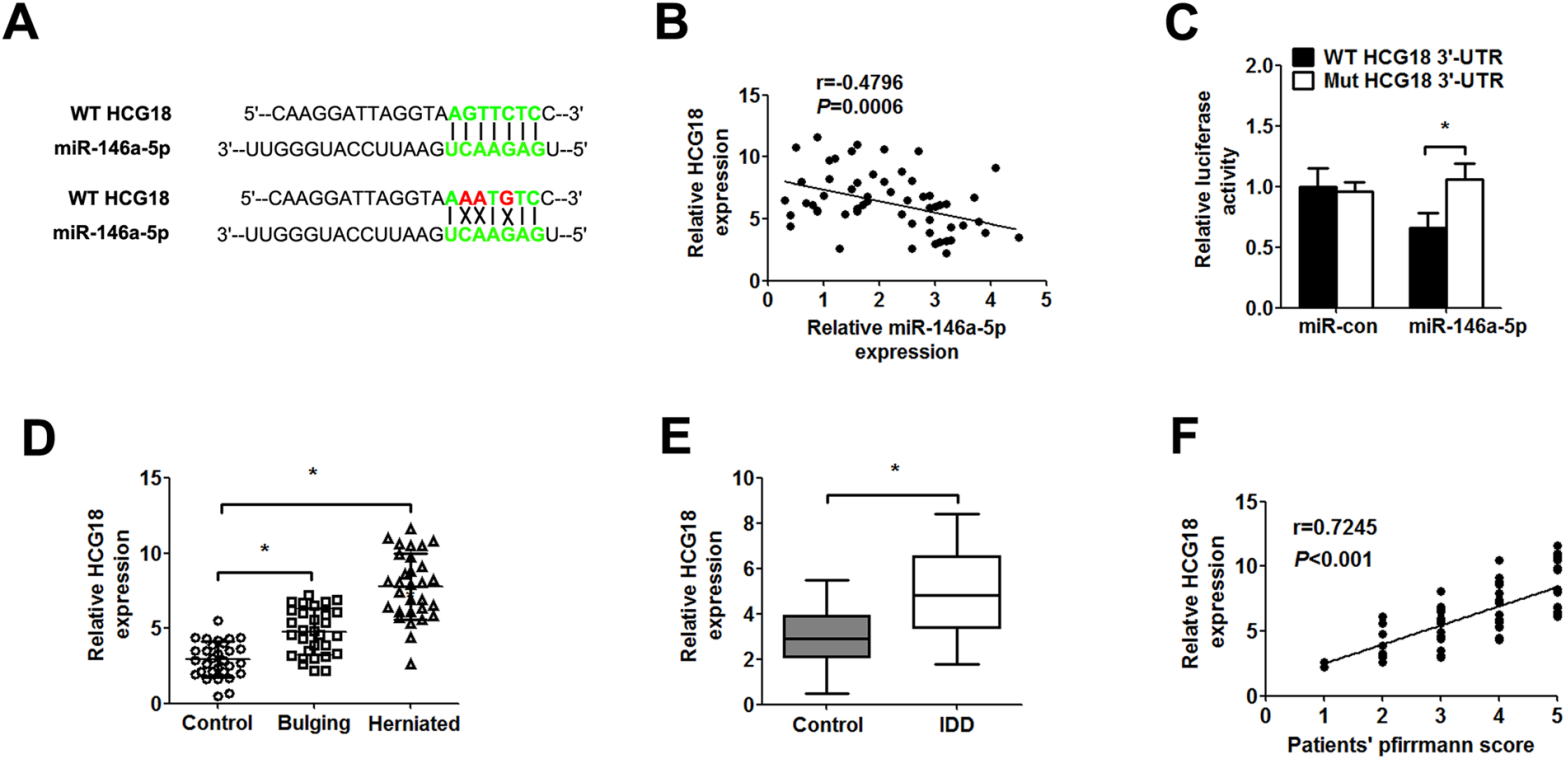
HCG18 is up-regulated in IDD patients. (**A**) Alignment of miR-146a-5p sequence with HCG18 and with HCG18 mutated at the putative binding site (37–43 region of HCG18, AGTTCTC). (**B**) The correlation between HCG18 level and miR-146a-5p expression was measured in NP tissues from the patients with bulging and herniated discs (n = 60). Spearman’s correlation coefficient r = 0.4796, *P* = 0.0006. (**C**) Luciferase reporter activity in NP cell was detected after co-transfection miR-con/miR-146a-5p mimics (25 nM) and luciferase vector (pmiR-GLo) containing the wild type/mutant HCG18 (100 ng/L). (**D**) HCG18 expression was measured by qPCR from control, bulging and herniated NP tissues (n = 90). (**E**) The relative expression of HCG18 in NP tissue from IDD patients and controls (n = 90). (**F**) The correlation between the expression of HCG18 and patients’ pfirrmann score (n = 60). Spearman’s correlation coefficient r = 0.7245, *P* = 0.0001. *P < 0.05.

Our previous study has demonstrated that miR-146a-5p inhibits the recruitment of macrophages and protects the NP cells from TNF-α-induced apoptosis by targeting TRAF6^14^. The bioinformatics method (starBase v2.0 algorithm, http://starbase.sysu.edu.cn/index.php) showed that HCG18 might function as miR-146a-5p sponge (Fig. 1A). The results indicated that there was a significant negative correlation between HCG18 and miR-146a-5p (Fig. 1B). To determine the direct binding between HCG18 and miR-146a-5p, we analyzed the potential binding sequence for miR-146a-5p in HCG18 and cloned the wild type/mutant fragments including the paired bases into a pmiR-GLO vector. The luciferase reporter assay indicated that miR-146a-5p mimics significantly reduced the luciferase activity of wild type pmirGLO-HCG18, but not of mutant pmirGLO-HCG18 in NP cells (Fig. 1C).

qPCR was performed to confirm the expression of HCG18 in the patients with spinal cord injury, bulging, herniated intervertebral disc and IDD. The expression of HCG18 was significantly up-regulated in the NP tissues from the patients with bulging or herniated discs compared with the controls (Fig. 1D). Similarly, degenerative NP tissues exhibited significantly higher expression of HCG18 when compared to the controls (Fig. 1E). In addition, the expression of HCG18 was positively correlated with disc degeneration grade (Fig. 1F).

### HCG18 regulates the proliferation and apoptosis of NP cells and recruitment of macrophages

**Figure 2:**
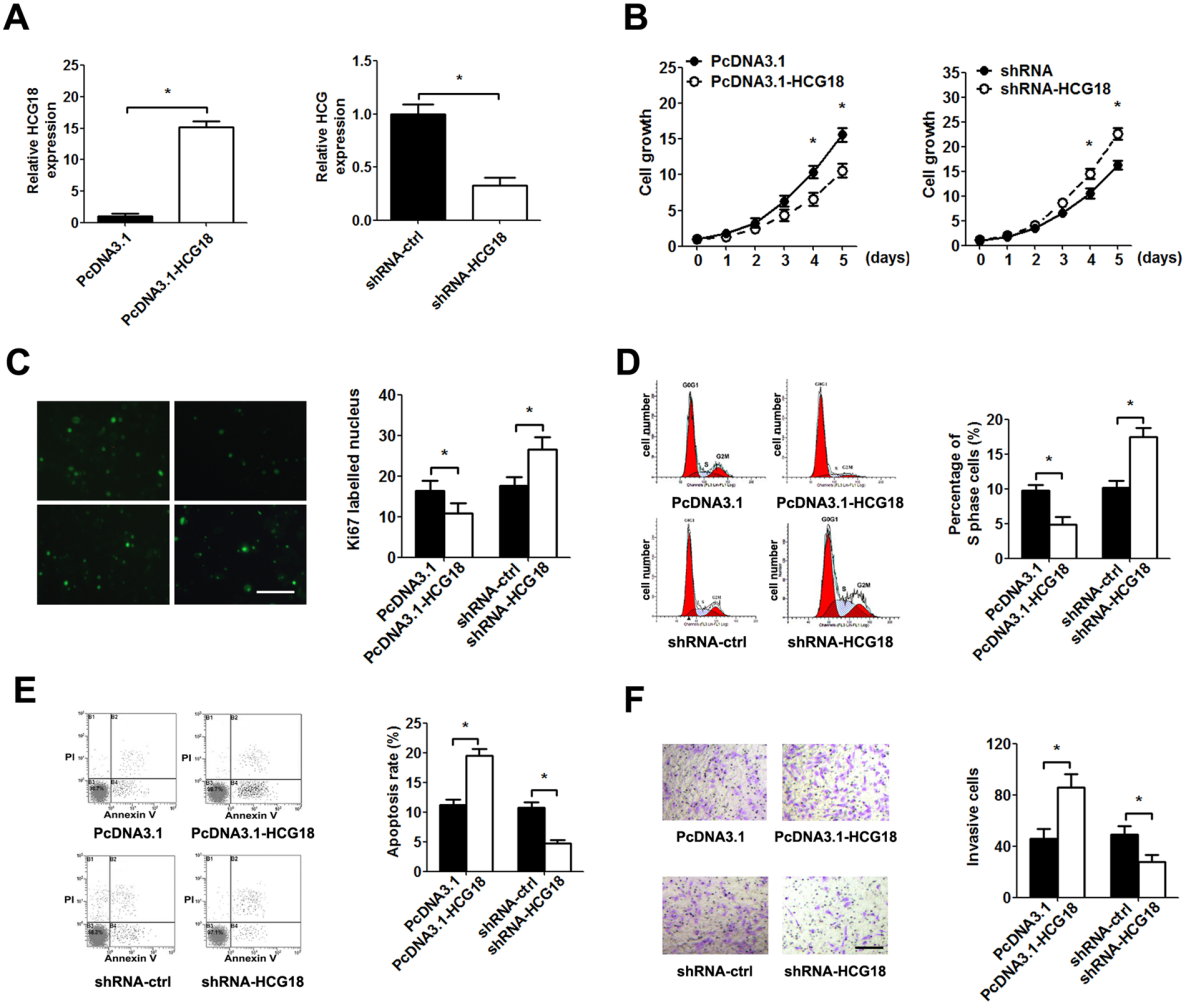
The effect of HCG18 on the proliferation, cell cycle, cell apoptosis in NP cells and macrophages recruitment. The NP cells were transfected with the HCG18 expression vector (100 ng/L), shRNA (25 nM) and control for 48 h. (**A**) The expression of HCG18 was detected by qPCR after transfection. (**B**) The CCK-8 assay was performed to examine proliferation of NP cells at 1, 2, 3, 4, 5 day. (**C**) Ki67 immunofluorescence staining was performed 96 h after transfection in NP cells. (**D**) The percentage of cell cycle in NP cells was detected by flow cytometry. (**E**) Apoptosis rate was analyzed using flow cytometry. (**F**) NP cells were treated with 10 ng/ml TNF-α for 48 h after transfection. Quantification of the number of invasive macrophages by counting 5 high-power fields in each chamber. *P < 0.05.

Given that the aberrant expression of HCG18 in degenerative NP tissues, we investigated the role of HCG18 on the proliferation and apoptosis of NP cells and recruitment of macrophages by transfection with HCG18 recombinant plasmid or shRNA (Fig. 2A). The MTT assay and Ki67 immunofluorescence staining indicated that overexpression of HCG18 in NP cells significantly inhibited the cell growth, whereas down-regulation of HCG18 increase the cell growth (Fig. 2B,C). Moreover, we assessed the cell cycle and apoptosis after transfection by flow cytometry. Overexpression of HCG18 in NP cells significantly decreased the percentage of S phase and induced apoptosis compared with the control group, while down-regulation of HCG18 exerted the opposite effect (Fig. 2D,E).

We also evaluated the role of HCG18 on TNF-α induced macrophage migration. The transwell migration assay indicated that up-regulation of HCG18 obviously elevated the number of migrated macrophages. In contrast, the number of migrated macrophages was significantly reduced compared with the controls after HCG18 expression was knockdown (Fig. 2F).

### Effect of HCG18 on osteogenic differentiation

**Figure 3:**
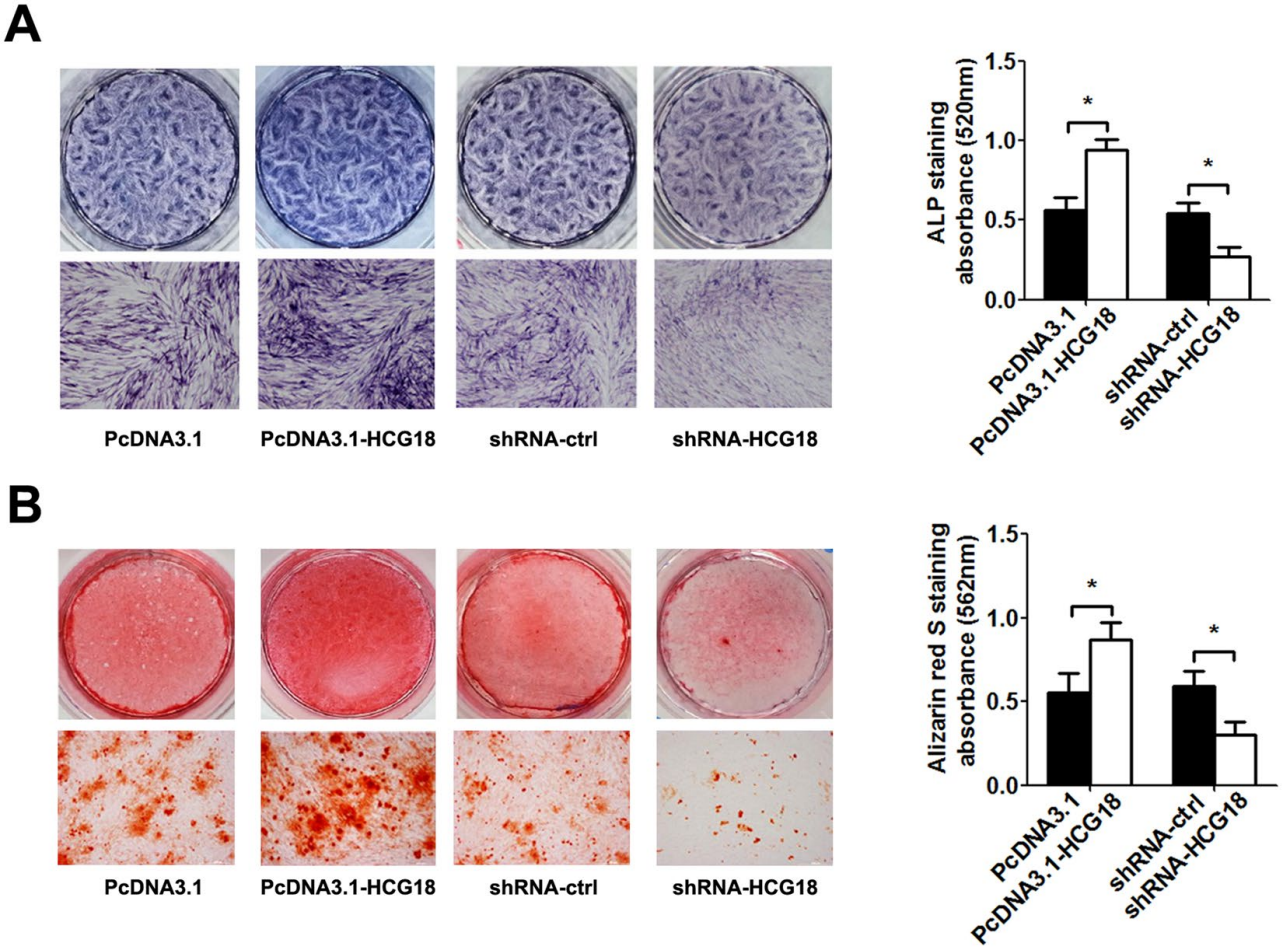
The impact of HCG18 on osteogenic differentiation of NP cell. DPSCs cells were transfected with the HCG18 expression vector, shRNA and control, and were incubated with osteogenic medium for 48 h. (**A**) Representative images of ALP staining 7 days after incubation. The absorbance value (520 nm) was quantified by Bio-reader. (**B**) Representative images of Alizarin red S staining 7 days after incubation. The absorbance value (562 nm) was quantified by Bio-reader. *P < 0.05.

Recent studies indicate that the process of calcification is associated with the process of IDD. We also investigated the effect of HCG18 on the osteogenic differentiation in NP cells by ALP staining and alizarin red staining. The results demonstrated that overexpression of HCG18 inhibited the osteogenic differentiation of NP cells. Conversely, as expected, knockdown of HCG18 promoted the osteogenic differentiation of NP cells (Fig. 3A,B).

### HCG18 exerts its biological effect by controlling miR-146a-5p/TRAF6/NFκB axis

**Figure 4:**
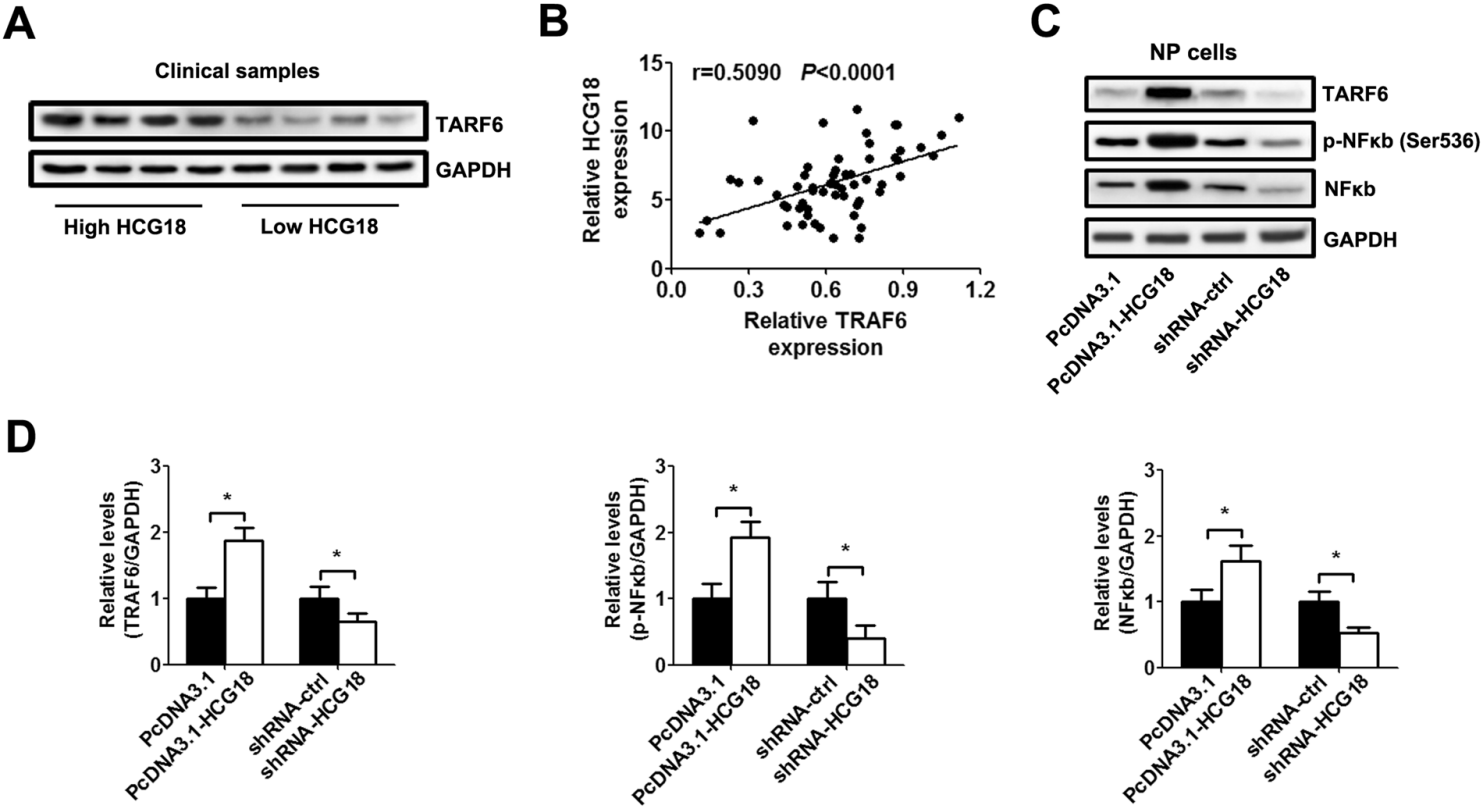
HCG18 regulated the TRAF6/NFκB signaling pathway. (**A**) The expression of TRAF6 was measured by western blot in NP tissues. (**B**) The correlation between HCG18 level and TRAF6 expression (n = 60). Spearman’s correlation coefficient r = 0.5090, *P* < 0.0001. (**C**) The NP cells were transfected with the HCG18 expression vector, shRNA and control for 48 h. The protein level of TRAF6, p-NFκB (Ser536) and NFκB was detected by western blot in NP cells. (**D**). The relative protein expression was calculated based on the densitometric analysis of band intensities shown in C. Full-length blots are presented in Supplementary Figures S1–S2. *P < 0.05.

A previous study indicated that miR-146a-5p directly inhibits TRAF6 gene expression via targeting its 3′UTR^14^. As expected, the expression of TRAF6 levels in NP tissues with high HCG18 level was significantly higher than those in NP tissue with low HCG18 level (Fig. 4A). The HCG18 level showed a positive correlation with TRAF6 expression in the NP tissues (Fig. 4B). *In vitro*, the expression of TRAF6, p-NFκB (Ser536) and NFκB was significantly enhanced after HCG18 was overexpressed in NP cells. However, the inhibition of HCG18 suppressed the expression of TRAF6 and NFκB (Fig. 4C,D).

**Figure 5:**
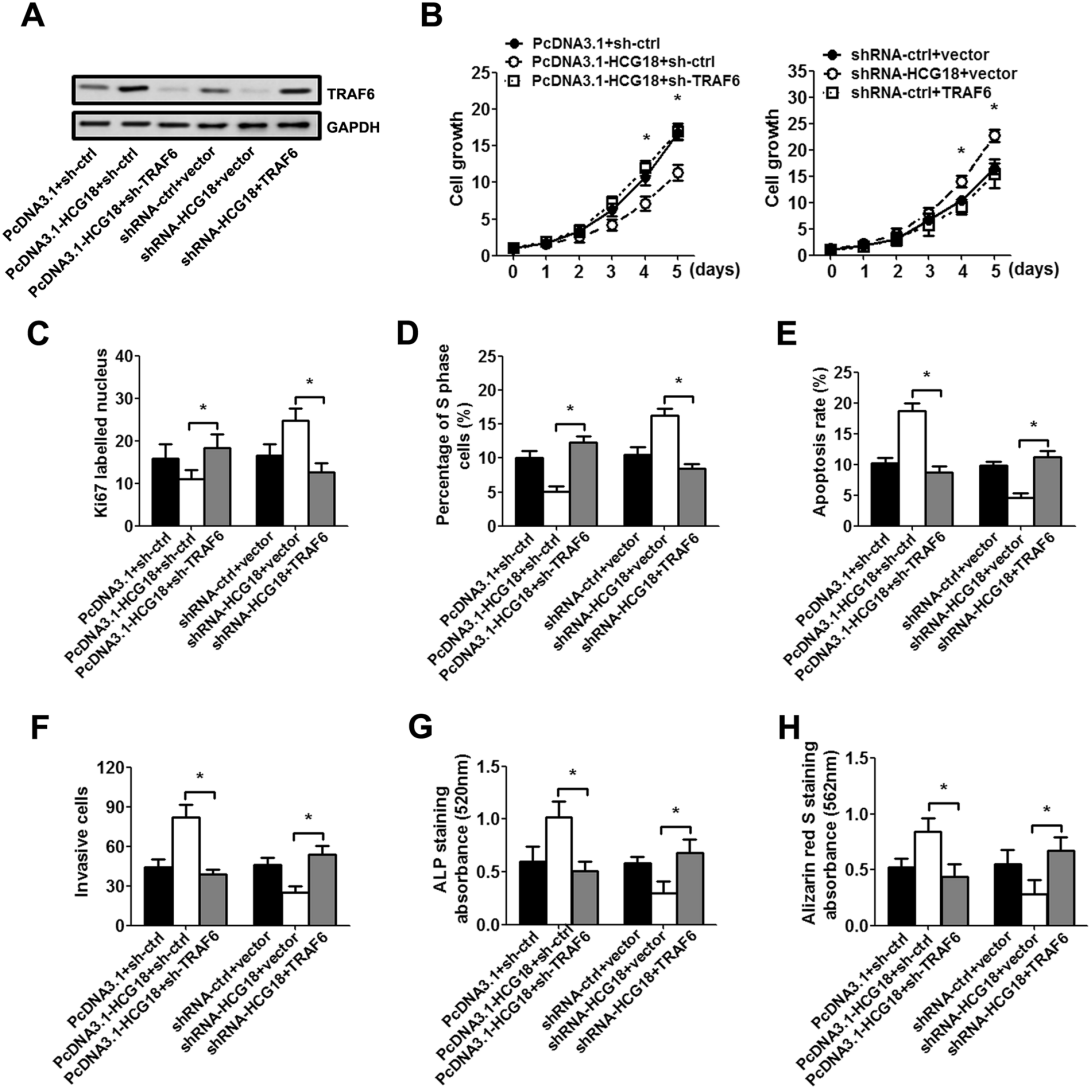
The biological effect of HCG18 on NP could be reversed by regulating the expression of TRAF6. NP cells were first infected with TRAF6 or TRAF6 shRNA virus (MOI = 50) for 48 h. Then, the cells were transfected with the HCG18 expression vector, shRNA and control for 48 h. (**A**) TRAF6 protein level was measured by western blot in NP tissues. Full-length blots are presented in Supplementary Figures S3. (**B**) Cell viability was measured with the CCK-8 assay. (**C**) Quantification of Ki67 labelled nucleus in NP cells. (**D**–**E**) Cell cycle and apoptosis rate were measured by flow cytometry. (**F**) Quantification of the number of invasive macrophages. (**G**–**H**) ALP staining and Alizarin red S staining were quantified using the absorbance value 7 days after incubation. *P < 0.05.

To investigate whether HCG18 exerts its biological effect by modulating the miR-146a-5p/TRAF6/NFκB axis, we repressed or restored the TRAF6 expression by TRAF6 overexpressing or interfering virus infection in NP cells. As expected, TRAF6 expression was decreased after TRAF6 interfering virus infection, and increased after TRAF6 overexpressing virus infection (Fig. 5A). The MTT assay and Ki67 immunofluorescence staining showed that down-regulation of TRAF6 abolished HCG18-mediated inhibition of the growth of NP cells (Fig. 5B,C). Similarly, infection with TRAF6 interfering virus increased the percentage of S phase, and reduced cell apoptosis in NP cells (Fig. 5D,E). Furthermore, HCG18-induced macrophage invasion (Fig. 5F) and osteogenic differentiation (Fig. 5G,H) were suppressed due to TRAF6 interfering virus infection. As expected, reintroduction of TRAF6 in low HCG18 expressing NP cells revealed a reverse result.

## Discussion

IDD is the most common chronic, prevalent and age-related degenerative musculoskeletal disorder^5^. LncRNAs have been shown to be differentially expressed in human degenerative NP tissue, and involved in the pathological processes of IDD, including inflammatory responses, apoptosis, proteoglycans degradation and ECM degeneration^3,8,15^. Therefore, the investigation of precise regulatory mechanism of lncRNA at the initial stage of IDD may be conductive to develop new diagnostic and therapeutic strategies for IDD and to improve the quality of life in IDD patients.

Emerging studies indicate that lncRNAs play an essential role in the regulation of gene expression by acting as miRNA sponges^13,16–18^. Previous our study suggested that miR-146a-5p is frequently down-regulated in human degenerative NP tissues (such as bulging and herniated discs)^14^. The dysregulation of miR-146a-5p can be attributed to inflammatory response in IDD. Bioinformatics prediction indicated that HCG18 may act as a miR-146a-5p sponge. The luciferase reporter assay confirmed that miR-146a-5p directly bound to wild type pmirGLO-HCG18, but not mutant pmirGLO-HCG18 in NP cells. In addition, we detected the expression of HCG18 in NP tissues from patients with bulging or herniated discs or IDD and spinal cord injury tissues. HCG18, a 2430-bp lncRNA that maps to chromosome 6p22.1, is expressed from NR_024052 locus. However, it is rarely reported especially the biological role of HCG18. The precise biological function and regulatory molecular mechanism of HCG18 are still unknown and need further investigation. Our result first demonstrated that the HCG18 level was up-regulated in NP tissues from patients with bulging or herniated discs. HCG18 expression was positively correlated with the disc degeneration grade and the hernia size, suggesting that HCG18 might act as a promoter in the development of IDD.

Recent studies have revealed some aberrantly expressed lncRNAs in human IDD^8^. In fact, lncRNAs have been regarded to function in IDD development^3^. The expression of the profiling of lncRNA reflects the true scenarios in human lumbar disc diseases and low back pain. In addition, current study demonstrated that the HCG18 inhibited the proliferation of NP cells and induced cell apoptosis, macrophage recruitment ability and osteogenic differentiation. These results are in accordance with our previous data, indicating that HCG18 is a novel booster in the progression of IDD by functioning as a competing endogenous miR-146a-5p. During the moderate and late stages of IDD, fibrocartilage-like tissue, bone formation, as well as nerve and blood vessels are found in the intervertebral disc^7,19^. A recent study shows that the presence of progenitor/stem cells can be attributed to this phenomenon^20–22^. NP cells have been demonstrated to be able to differentiate into cartilage, fibrocartilage cells, osteoblasts, neurons, and endothelial cells in response to different stimuli^23^. Our data first indicated that HCG18 promotes the osteogenic differentiation which may contribute to chronic low-back pain and low quality of life in IDD patients.

To elucidate the molecular mechanism of HCG18 in IDD progression, we validated the TRAF6/NFκB signaling pathway which plays an important role in inflammatory response and pro-inflammatory cytokines release^24,25^. We found that HCG18 level was positively associated with TRAF6 expression in NP tissue. Overexpression of HCG18 activated the TRAF6/NFκB signaling pathway, leading to the recruitment of macrophages and apoptosis of NP cells in intervertebral discs. A recent study suggested that overexpression of miR-146a significantly decreased the levels of pro-inflammatory cytokines, including IL-1b, TNF-α, and IL-6 by targeting the TARF6/NFκB pathway. It is indicated that miR-146a ameliorates inflammation via the TRAF6/NF-kB pathway in intervertebral disc cells^26,27^. Our results lend credence to the previous study suggesting that HCG18, acting as a miR-146a-5p sponge, accelerate IDD progression via the miR-146a-5p/TARF6/NFκB axis. Moreover, down-regulation of TRAF6 abolished HCG18-mediated effect on the proliferation and apoptosis of NP cells, macrophage recruitment and osteogenic differentiation. These results imply that HCG18/miR-146a-5p/TARF6/NFκB axis exert a critical function in the pathogenesis of IDD.

In conclusion, our results suggested that HCG18 acts a sponge of miR-146a-5p in NP cells, and the HCG18 level was up-regulated in IDD. Furthermore, HCG18 plays a crucial role in the proliferation and apoptosis of NP cells, macrophage recruitment, and osteogenic differentiation via the miR-146a-5p/TARF6/NFκB axis. Taken together, HCG18 represents a novel early diagnostic marker of IDD and an efficient anabolic strategy for IDD patients. In order to clarify the potential of clinical translation of HCG18 in IDD, an *in vivo* murine IDD model was used to explore the clinical application of HCG18 in the future work.

## Materials and Methods

### Patients and tissue samples

The human lumber NP specimens were obtained from 120 patients with IDD (n = 30), bulging discs (n = 30), herniated discs (n = 30), and spinal cord injury (control, n = 30) between August 2010 and June 2016 from the Department of orthopedics, Changzheng hospital, Second Military Medical University (Shanghai, China). All included patients had typically clinical symptoms, and the degree of IDD was evaluated on magnetic resonance imaging (MRI) scan according to a modified pfirrmann grading classification. The specimens were first isolated within 30 min, and then divided into two parts (frozen in liquid nitrogen for store or isolated for NP cell culture). This study (No. SMMU2010023) was approved by the Ethics Review Board of Changzheng hospital, Second Military Medical University. Written informed consent was gathered from all participants. In this study, all methods were performed in accordance with the relevant guidelines and regulations.

### RNA Extraction and lncRNAs expression assay

Total RNA from NP tissue and cell were extracted using Trizol reagent (Invitrogen, USA) according to the manufacturer’s instructions. The RNA concentration was measured using a NanoDrop ND-1000 spectrophotometer (Thermo Fisher Scientific, Inc., Wilmington, DE, USA). Then, the agarose gel electrophoresis was used to determine RNA integrity. cDNA was generated using the Reverse Transcription Kit (Invitrogen, Carlsbad, CA, USA). Real-time quantitative polymerase chain reactions (RT-qPCR) were performed using the SYBR Green PCR kit protocol in the 7000 Sequence Detection System (Applied Biosystems, Carlsbad, CA, USA). U6 snRNA was used for normalization, and the relative lncRNA expression was calculated using the 2^−ΔΔCt^ method.

### Culture of NP cells

The NP tissue was gently separated from the disc under aseptic condition, washed by phosphate-buffered saline (PBS) for three times, and then cut into small pieces with ophthalmic scissors. Subsequently, tissue was digested with PBS containing 0.025% type II collagenase (Invitrogen, Carlsbad, CA, USA) for 4 h followed by filtration and centrifugation at 500 g for 10 min. The supernatant was removed, the NP cells were seeded into culture dishes in DMEM/F12 medium (GE Healthcare Life Sciences, Logan, Utah, USA) containing 15% fetal bovine serum (FBS, Gibco, Shanghai, China) and 100 U/ml streptomycin/penicillin under 5% CO_2_, saturated humidity at 37 °C for 3 days. The culture medium was changed three times a week, and NP cells were subcultured at a ratio of 1:3 after reaching 80% confluence.

### Plasmids and cell transfection

For HCG18 overexpression, the complementary DNA encoding of HCG18 was PCR-amplified by PrimeSTAR HS DNA Polymerase (TaKaRa, Dalian, China), and cloned into the pcDNA3.1 vector (Invitrogen, Carlsbad, CA, USA). LncRNA shRNA (5′-TTGGCTTCAGTCCTGTTCATCAG-3′) and miRNA mimics were synthesized by GenephPharma (Shanghai, China). 5 × 10^4^ cells were seeded into 24-well dishes with 1 ml/well medium. The diluted transfections were mixed carefully with Lipofectamine 3000 (Invitrogen, Carlsbad, CA, USA) was used for transfection according to the manufacturer’s instructions when the cell density reached 70%. Cell were harvested for subsequent analyses 48 h after transfection.

### Luciferase reporter assays

For the luciferase reporter assay, full length sequence of HCG18 (2430-bp, LNCipedia gene ID: lnc-TRIM 26–2) was amplified using PCR and subcloned into the pmirGLO vector (Promega, Madison, WI, USA). The QuikChange® Site-Directed Mutagenesis Kit (Stratagene, Hangzhou, Zhejiang, China) was used for recombinant plasmid point mutation. NP cells were cotransfected with wild type pmirGLO-HCG18/mutant pmirGLO-HCG18 and miR-con/miR-146a-5p using Lipofectamine 2000 (Invitrogen, Carlsbad, CA, USA). Forty-eight hours after transfection, cells were harvested using passive lysis buffer followed by the measurement of luciferase activities with the Dual-Luciferase Reporter Assay System (E2920, Promega, USA).

### Cell proliferation assays

The Cell Counting Kit-8 (CCK-8, Dojindo, Kumamoto, Japan) was utilized to detect the proliferation of cells following the manufacturer’s instructions. Briefly, the cells were seeded onto 96-well plates (1 × 10^3^ cells/well), and the cell viability was measured at the indicated time (1, 2, 3, and 4 days) with absorbance at 450 nm using a ELx 800 Microplate Reader (Bio-Tek Instruments Inc., Winooski, VT).

### Immunofluorescence staining

NP cells were rinsed with PBS for three times, fixed with 4% formaldehyde for 15 min, and permeabilized with 0.3% Triton-X100 (Sigma, CO, USA) for 10 min. Subsequently, the cells were blocked with 6% BSA for 1 h and incubated with primary antibody against Ki67 (dilution, 1:200; Rabbit polyclonal antibody, Abcam, Shanghai, China) overnight at 4 °C. Then, cells were incubated with Alexa Flour 488 labeled goat anti-mouse IgG (Invitrogen 1:200; Invitrogen, OR, USA) for 1 h at room temperature. Cells were analyzed using the Olympus DP72 fluorescence microscope (Olympus, Tokyo, Japan).

### Flow cytometry analysis

Cell cycle and apoptosis were measured by flow cytometry FACSDiva 6.1.1 (Becton Dickinson). For cell cycle, the cells were gathered, and fixed with 70% ethanol at −20 °C overnight. Then, cells were treated with 20 µg/ml RNaseA (Sigma-Aldrich, St. Louis, MO, USA) and incubated with 50 µg/ml propidium iodide (Sigma-Aldrich, St. Louis, MO, USA) for 30 min at 37 °C. The Annexin-V fluorescein isothiocyanate (FITC) Apoptosis Kit (BD Biosciences, Sparks, MD) was used to assess the cell apoptosis according to the manufacturer’s introduction.

### Transwell invasion assays

The RAW264.7 macrophage cell line, purchased from the American Type Culture Collection (ATCC, Rockville, MD, USA), was cultured in DMEM supplemented with 10% FBS and 100 U/ml penicillin/streptomycin in a cell culture incubator at 37 °C and 5% CO_2_. The macrophages transwell invasion assay was performed as previously described. Briefly, a total of 1 × 10^6^ macrophages were seeded in the upper wells of chambers (8-µm pore size; Corning Inc., Corning, NY, USA) with DMEM containing 0.1% FBS. 1 × 10^6^ NP cells in the lower well were grown in 1 ml of DMEM containing 10 ng/ml TNF-α (Sigma, St. Louis, MO, USA). Twenty hours later, the invasive cells were stained with 2% crystal violet (Sigma), and counted in 5 high-power fields under the microscopic fields.

### Western Blot

The cells were lysed using the RIPA buffer (Sigma, St. Louis, MO, USA). Total protein was separated by 12% sodium dodecyl sulphate-polyacrylamide (SDS-PAGE) electrophoresis and transferred to nitrocellulose membranes (Millipore, Billerica, MA, USA), followed by incubation with TRAF6, p-NFκB (Ser536), NFκB and GAPDH (Cell Signaling Technology, Boston, USA) overnight at 4 °C. After being rinsed thrice, the membranes were further incubated with a HRP-conjugated anti-IgG for 1 h at 37 °C. The protein was detected using an ECL system (Amersham Pharmacia, Piscataway, NJ, USA) were analyzed using the Quantity One software (BIO-RAD, USA).

### Alkaline phosphatase (ALP) staining and alizarin red staining

For osteogenic differentiation, NP cells were cultured in osteogenic-inducing medium as previously described^28^. After 7-day osteogenic differentiation, ALP activity was evaluated with the ALP staining kit (Biyuntian Biotech Co., Ltd., Shanghai, China). The mineralised matrix in NP cells was detected using Alizarin red (Sigma–Aldrich, St Louis, MO) staining. Calcium nodules were imaged under a light microscope and quantified spectroscopically at 562 nm with a microplate reader (BIO-TEK, Winooski, VT, USA).

### TRAF6 viral infection

Human TRAF6 virus and TRAF6 shRNA were constructed as our previous study described^14^. All assays were conducted 48 h after viral infection or shRNA transfection.

### Statistical analysis

Statistical analysis was performed using the StatView 5.0 software (SAS Institute, Cary, NC). All the data were expressed as mean ± standard deviation (SD). Student’s t-tests were performed to compare the differences between two groups, and differences among three or more groups were evaluated by one-way ANOVA. The correlation between the expression of HCG18 and miR-146a-5p level, duration of symptoms and TRAF6 expression was determined by Spearman’s correlation analysis. All experiments were performed independently in triplicate. *P* < 0.05 was considered statistically significant.

## Electronic supplementary material

Supplementary data
