# Supplementary material for: Long non-coding HCG18 promotes intervertebral disc degeneration by sponging miR-146a-5p and regulating TRAF6 expression
Source: Sci Rep. 2017 Oct 16;7:13234. doi: 10.1038/s41598-017-13364-6 (PMC5643303; doi:10.1038/s41598-017-13364-6)
Supplement: Supplementary file 1 — Supplementary data [file 41598_2017_13364_MOESM1_ESM.doc]

**Long non-coding HCG18 promotes intervertebral disc degeneration by sponging miR-146a-5p and regulating TRAF6 expression**

Yanhai Xi^1^,*, Tingwang Jiang^2^,*, Weiheng Wang^1^, Jiangming Yu^1^, Yang Wang^1^, Xueming Wu^1^, Yunfei He^1^

^1^Department of orthopedics, Changzheng hospital, Second Military Medical University, Shanghai 200003, China

^2^Department of Immunology and Microbiology, Institution of Laboratory Medicine of Changshu, Changshu 215500, Jiangsu, China

*These authors contributed equally to this work.

Correspondence to: Yanhai Xi, Department of Orthopaedics, Changzheng Hospital, Second Military Medical University, 415 Fengyang Road, Shanghai 200003, China.

E-mail: xiyanhai1778@qq.com Tel: +86-021-81886999


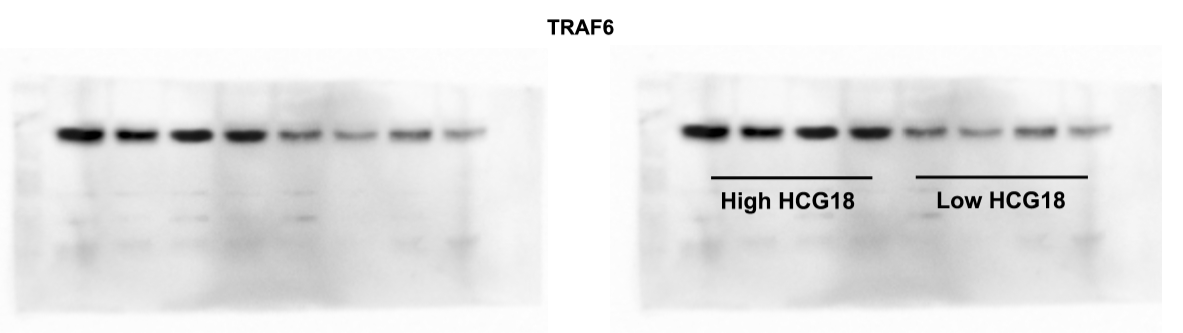


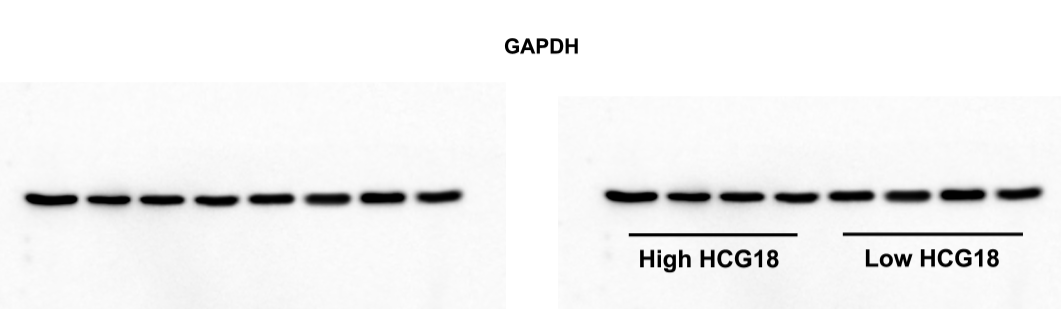


**Figure S1.** The Full-length immunblots as shown in Figure 4A.


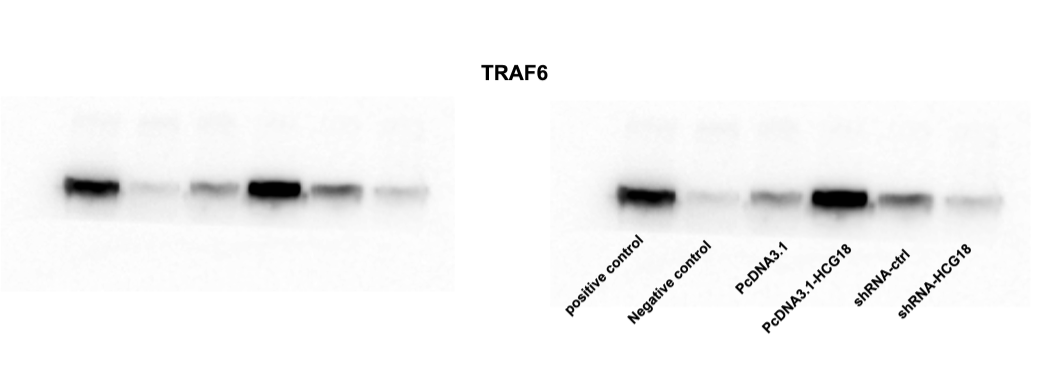


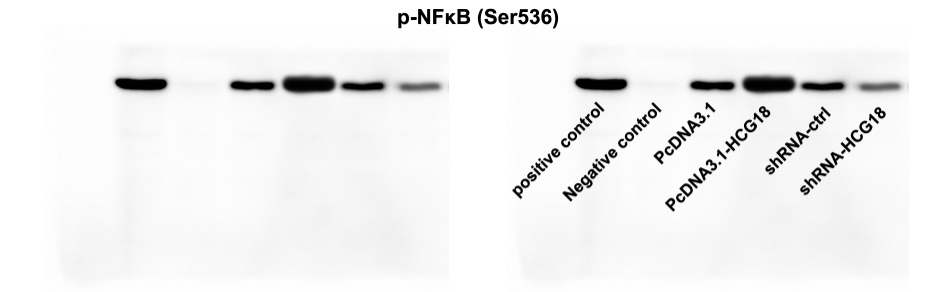


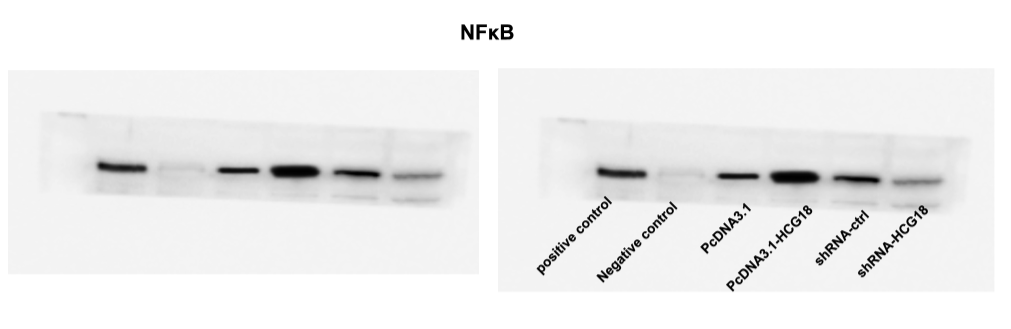


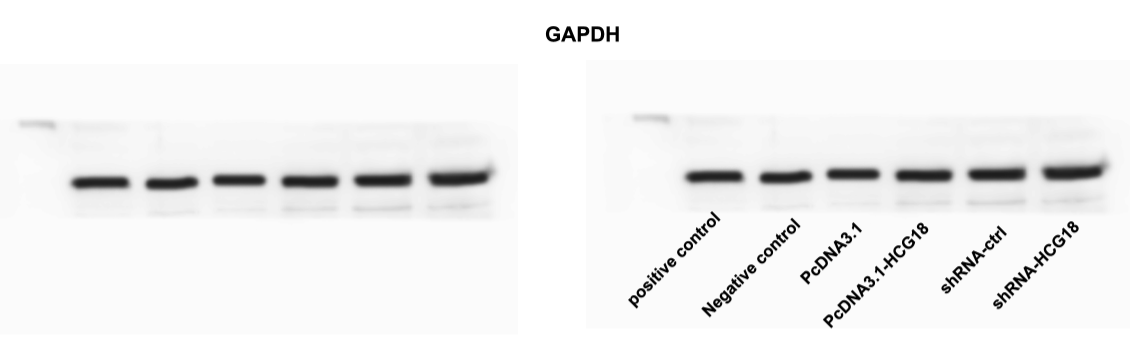


**Figure S2.** The Full-length immunblots as shown in Figure 4C.


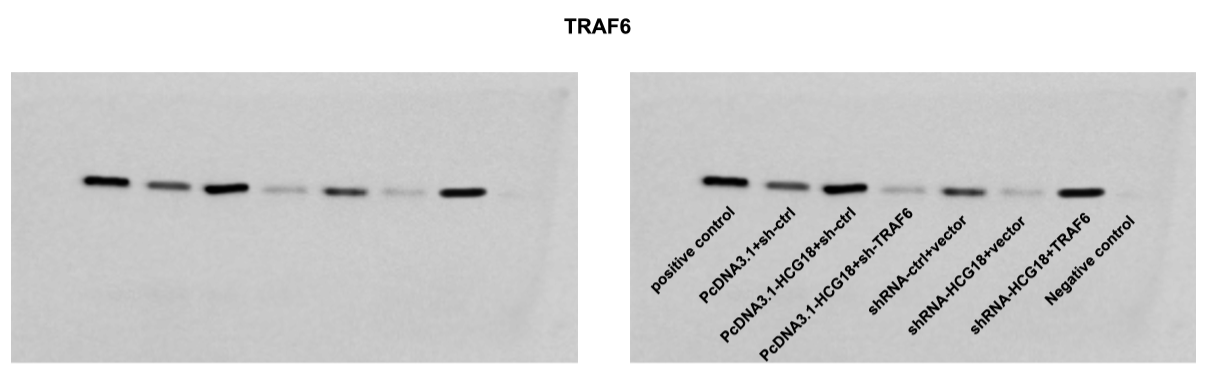


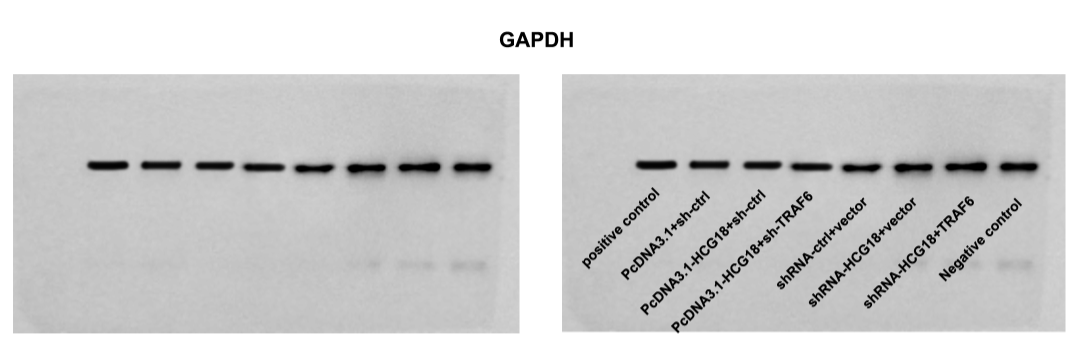


**Figure S3.** The Full-length immunblots as shown in Figure 5A.
